# Author Correction: The European Reference Genome Atlas: piloting a decentralised approach to equitable biodiversity genomics

**DOI:** 10.1038/s44185-024-00065-3

**Published:** 2024-10-15

**Authors:** Ann M. Mc Cartney, Giulio Formenti, Alice Mouton, Diego De Panis, Luísa S. Marins, Henrique G. Leitão, Genevieve Diedericks, Joseph Kirangwa, Marco Morselli, Judit Salces-Ortiz, Nuria Escudero, Alessio Iannucci, Chiara Natali, Hannes Svardal, Rosa Fernández, Tim De Pooter, Geert Joris, Mojca Strazisar, Jonathan M. D. Wood, Katie E. Herron, Ole Seehausen, Phillip C. Watts, Felix Shaw, Robert P. Davey, Alice Minotto, José M. Fernández, Astrid Böhne, Carla Alegria, Tyler Alioto, Paulo C. Alves, Isabel R. Amorim, Jean-Marc Aury, Niclas Backstrom, Petr Baldrian, Laima Baltrunaite, Endre Barta, Bertrand BedHom, Caroline Belser, Johannes Bergsten, Laurie Bertrand, Helena Bilandija, Mahesh Binzer-Panchal, Iliana Bista, Mark Blaxter, Paulo A. V. Borges, Guilherme Borges Dias, Mirte Bosse, Tom Brown, Rémy Bruggmann, Elena Buena-Atienza, Josephine Burgin, Elena Buzan, Alessia Cariani, Nicolas Casadei, Matteo Chiara, Sergio Chozas, Fedor Čiampor, Angelica Crottini, Corinne Cruaud, Fernando Cruz, Love Dalen, Alessio De Biase, Javier del Campo, Teo Delic, Alice B. Dennis, Martijn F. L. Derks, Maria Angela Diroma, Mihajla Djan, Simone Duprat, Klara Eleftheriadi, Philine G. D. Feulner, Jean-François Flot, Giobbe Forni, Bruno Fosso, Pascal Fournier, Christine Fournier-Chambrillon, Toni Gabaldon, Shilpa Garg, Carmela Gissi, Luca Giupponi, Jessica Gomez-Garrido, Josefa González, Miguel L. Grilo, Björn Grüning, Thomas Guerin, Nadege Guiglielmoni, Marta Gut, Marcel P. Haesler, Christoph Hahn, Balint Halpern, Peter W. Harrison, Julia Heintz, Maris Hindrikson, Jacob Höglund, Kerstin Howe, Graham M. Hughes, Benjamin Istace, Mark J. Cock, Franc Janžekovič, Zophonias O. Jonsson, Sagane Joye-Dind, Janne J. Koskimäki, Boris Krystufek, Justyna Kubacka, Heiner Kuhl, Szilvia Kusza, Karine Labadie, Meri Lähteenaro, Henrik Lantz, Anton Lavrinienko, Lucas Leclère, Ricardo Jorge Lopes, Ole Madsen, Ghislaine Magdelenat, Giulia Magoga, Tereza Manousaki, Tapio Mappes, Joao Pedro Marques, Gemma I. Martinez Redondo, Florian Maumus, Shane A. McCarthy, Hendrik-Jan Megens, Jose Melo-Ferreira, Sofia L. Mendes, Matteo Montagna, Joao Moreno, Mai-Britt Mosbech, Mónica Moura, Zuzana Musilova, Eugene Myers, Will J. Nash, Alexander Nater, Pamela Nicholson, Manuel Niell, Reindert Nijland, Benjamin Noel, Karin Noren, Pedro H. Oliveira, Remi-Andre Olsen, Lino Ometto, Rebekah A. Oomen, Stephan Ossowski, Vaidas Palinauskas, Snaebjorn Palsson, Jerome P. Panibe, Joana Pauperio, Martina Pavlek, Emilie Payen, Julia Pawlowska, Jaume Pellicer, Graziano Pesole, Joao Pimenta, Martin Pippel, Anna Maria Pirttilä, Nikos Poulakakis, Jeena Rajan, Rúben M.C. Rego, Roberto Resendes, Philipp Resl, Ana Riesgo, Patrik Rodin-Morch, Andre E. R. Soares, Carlos Rodriguez Fernandes, Maria M. Romeiras, Guilherme Roxo, Lukas Rüber, Maria Jose Ruiz-Lopez, Urmas Saarma, Luis P. da Silva, Manuela Sim-Sim, Lucile Soler, Vitor C. Sousa, Carla Sousa Santos, Alberto Spada, Milomir Stefanovic, Viktor Steger, Josefin Stiller, Matthias Stöck, Torsten H. Struck, Hiranya Sudasinghe, Riikka Tapanainen, Christian Tellgren-Roth, Helena Trindade, Yevhen Tukalenko, Ilenia Urso, Benoit Vacherie, Steven M. Van Belleghem, Kees Van Oers, Carlos Vargas-Chavez, Nevena Velickovic, Noel Vella, Adriana Vella, Cristiano Vernesi, Sara Vicente, Sara Villa, Olga Vinnere Pettersson, Filip A. M. Volckaert, Judit Voros, Patrick Wincker, Sylke Winkler, Claudio Ciofi, Robert M. Waterhouse, Camila J. Mazzoni

**Affiliations:** 1 Genomics Institute, University of California, Santa Cruz, CA USA; 2 The Vertebrate Genome Laboratory, The Rockefeller University, New York, NY USA; 3 Department of Biology, University of Florence, Sesto Fiorentino, Italy; 4 InBios-Conservation Genetics Laboratory, University of Liege, Liege, Belgium; 5 Leibniz Institut für Zoo und Wildtierforschung, Berlin, Germany; 6 Berlin Center for Genomics in Biodiversity Research, Berlin, Germany; 7 Department of Biology, University of Antwerp, Antwerp, Belgium; 8 Institute of Zoology, University of Cologne, Cologne, Germany; 9 Department of Chemistry, Life Sciences and Environmental Sustainability, University of Parma, Parma, Italy; 10 Institute of Evolutionary Biology (CSIC-Universitat Pompeu Fabra), Barcelona, Spain; 11 Naturalis Biodiversity Center, Leiden, The Netherlands; 12 Neuromics Support Facility, VIB Center for Molecular Neurology, VIB, Antwerp, Belgium; 13 Neuromics Support Facility, Department of Biomedical Sciences, University of Antwerp, Antwerp, Belgium; 14 Tree of Life, Wellcome Sanger Institute, Hinxton, Cambridge, UK; 15 School of Biology and Environmental Science, University College Dublin, Belfield, Ireland; 16 Aquatic Ecology & Evolution, Institute of Ecology & Evolution, University of Bern, Bern, Switzerland; 17 Department of Fish Ecology & Evolution, Eawag, Kastanienbaum, Switzerland; 18 Department of Biological and Environmental Science, University of Jyvaskyla, Jyvaskyla, Finland; 19 The Earlham Institute, Norwich Research Park, Norwich, UK; 20 Digital Science, London, UK; 21 Barcelona Supercomputing Center; Spanish National Bioinformatics Institute, ELIXIR Spain, Getafe, Spain; 22 Leibniz Institute for the Analysis of Biodiversity Change, Museum Koenig Bonn, Bonn, Germany; 23 CE3C—Centre for Ecology, Evolution and Environmental Changes & CHANGE—Global Change and Sustainability Institute, Faculdade de Ciências, Universidade de Lisboa, Campo Grande, Lisboa Portugal; 24 Centro Nacional de Análisis Genómico (CNAG), Barcelona, Spain; 25 Universitat de Barcelona (UB), Barcelona, Spain; 26 CIBIO, Centro de Investigacao em Biodiversidade e Recursos Geneticos, InBIO Laboratorio Associado, Universidade do Porto, Vairao, Portugal; 27 Departamento de Biologia, Faculdade de Ciencias, Universidade do Porto, Porto, Portugal; 28 BIOPOLIS Program in Genomics, Biodiversity and Land Planning, CIBIO, Campus de Vairao, Vairao, Portugal; 29 University of the Azores, cE3c—Centre for Ecology, Evolution and Environmental Changes, Azorean Biodiversity Group, CHANGE—Global Change and Sustainability Institute, Rua Capitão João d´Ávila, Pico da Urze, Angra do Heroísmo, Portugal; 30 Génomique Métabolique, Genoscope, Institut François Jacob, CEA, CNRS, Univ Evry, Université Paris-Saclay, Evry, France; 31 Evolutionary Biology Program, Department of Ecology and Genetics, Uppsala University, Uppsala, Sweden; 32 Institute of Microbiology of the Czech Academy of Sciences, Praha, Czech Republic; 33 Nature Research Centre, Debrecen, Hungary; 34 Institute of Biochemistry and Molecular Biology, Faculty of Medicine, University of Debrecen, Debrecen, Hungary; 35 Institut de Systematique, Evolution, Biodiversite, Museum National d Histoire Naturelle, CNRS, Sorbonne Université, EPHE, Université des Antilles, Paris, France; 36 Department of Zoology, Swedish Museum of Natural History, Stockholm, Sweden; 37 Department of Zoology, Faculty of Science, Stockholm University, Stockholm, Sweden; 38 Genoscope, Institut François Jacob, CEA, Université Paris-Saclay, Evry, France; 39 Ruder Boskovic Institute, Zagreb, Croatia; 40 SciLifeLab, Solna, Sweden; 41 Uppsala University, Uppsala, Sweden; 42 National Bioinformatics Infrastructure Sweden, Uppsala, Sweden; 43 Senckenberg Research Institute, Frankfurt, Germany; 44 LOEWE Centre for Translational Biodiversity Genomics, Frankfurt, Germany; 45 Wellcome CRUK Gurdon Institute, University of Cambridge, Cambridge, UK; 46 VU University Amsterdam, Amsterdam, The Netherlands; 47 Animal Breeding & Genomics, Wageningen University & Research, Wageningen, The Netherlands; 48 Wageningen University & Research, Wageningen, The Netherlands; 49 Max Planck Institute of Molecular Cell Biology and Genetics, Dresden, Germany; 50 DRESDEN concept Genome Center, Dresden, Germany; 51 Interfaculty Bioinformatics Unit and Swiss Institute of Bioinformatics, University of Bern, Bern, Switzerland; 52 Institute of Medical Genetics and Applied Genomics, University of Tubingen, Tubingen, Germany; 53 NGS Competence Center Tubingen, Tubingen, Germany; 54 European Molecular Biology Laboratory, European Bioinformatics Institute, Wellcome Genome Campus, Hinxton, Cambridge, UK; 55 University of Primorska, Faculty of Mathematics, Natural Sciences and Information Technologies, Koper, Slovenia; 56 Faculty of Environmental Protection, Velenje, Slovenia; 57 Department of Biological, Geological and Environmental Sciences, Alma Mater Studiorum Universitá di Bologna, Bologna, Italy; 58 Department of Biosciences, Università degli Studi di Milano, Milan, Italy; 59 Institute of Biomembranes, Bioenergetics and Molecular Biotechnologies, Consiglio Nazionale delle Ricerche, Bari, Italy; 60 Sociedade Portuguesa de Botânica, Lisbon, Portugal; 61 Department of Biodiversity and Ecology, Plant Science and Biodiversity Centre Slovak Academy of Sciences, Bratislava, Slovakia; 62 Department of Zoology, Stockholm University, Stockholm, Sweden; 63 Department of Bioinformatics and Genetics, Swedish Museum of Natural History, Stockholm, Sweden; 64 Centre for Palaeogenetics, Stockholm, Sweden; 65 Department of Biology and Biotechnologies, Sapienza University of Rome, Rome, Italy; 66 University of Ljubljana, Biotechnical Faculty, Department of Biology, Ljubljana, Slovenia; 67 University of Namur, Department of Biology, URBE, ILEE, Namur, Belgium; 68 Department of Biology and Ecology, University of Novi Sad, Novi Sad, Serbia; 69 Eawag Swiss Federal Institute of Aquatic Science and Technology, Department of Fish Ecology & Evolution, Kastanienbaum, Switzerland; 70 Department of Organismal Biology, Universite libre de Bruxelles, Brussels, Belgium; 71 Department of Biosciences, Biotechnology and Environment, University of Bari Aldo Moro, Bari, Italy; 72 Groupe de Recherche et d Etude pour la Gestion de l Environnement, Villandraut, France; 73 Barcelona Supercomputing Centre (BSC), Barcelona, Spain; 74 Institute for Research in Biomedicine (IRB), Barcelona, Spain; 75 Catalan Institution for Research and Advanced Studies (ICREA), Barcelona, Spain; 76 CIBERINFEC, Instituto Carlos III, Barcelona, Spain; 77 NNF Center for Biosustainability, Technical University of Denmark, Kongens Lyngby, Denmark; 78 CoNISMa, Consorzio Nazionale Interuniversitario per le Scienze del Mare, Roma, Italy; 79 Centre of Applied Studies for the Sustainable Management and Protection of Mountain Areas CRC Ge.S.Di.Mont., University of Milan, Milan, Italy; 80 Department of Agricultural and Environmental Sciences-Production, Landscape and Agroenergy DiSAA, University of Milan, Milan, Italy; 81 Marine and Environmental Sciences Centre, Aquatic Research Network, Instituto Universitário de Ciências Psicológicas, Sociais e da Vida, Lisboa, Portugal; 82 Egas Moniz Center for Interdisciplinary Research (CiiEM), Egas Moniz School of Health & Science, Caparica, Portugal; 83 Bioinformatics Group, Department of Computer Science, Albert-Ludwigs-University Freiburg, Freiburg, Germany; 84 University of Cologne, Cologne, Germany; 85 Department of Biology, University of Graz, Graz, Austria; 86 MME BirdLife Hungary, Budapest, Hungary; 87 Doctoral School of Biology, Department of Systematic Zoology and Ecology, Institute of Biology, ELTE Eotvos Lorand University, Budapest, Hungary; 88 HUN-REN-ELTE-MTM Integrative Ecology Research Group, Budapest, Hungary; 89 Department of Zoology, Institute of Ecology and Earth Sciences, University of Tartu, Tartu, Estonia; 90 Institute of Life and Environmental Sciences, University of Iceland, Reykjavik, Iceland; 91 UCD Conway Institute, University College Dublin, Belfield, Ireland; 92 Algal Genetics Group, UMR 8227, CNRS, Sorbonne Universite, UPMC University Paris 06, Paris, France; 93 France Integrative Biology of Marine Models, Station Biologique de Roscoff, Roscoff, France; 94 University of Maribor, Faculty of Natural Sciences and Mathematics, Maribor, Slovenia; 95 Department of Ecology and Evolution, University of Lausanne, Lausanne, Switzerland; 96 Swiss Institute of Bioinformatics, Lausanne, Switzerland; 97 Ecology and Genetics Research Unit, University of Oulu, Oulu, Finland; 98 Slovenian Museum of Natural History, Ljubljana, Slovenia; 99 Science and Research Centre Koper, Koper, Slovenia; 100 Museum and Institute of Zoology, Polish Academy of Sciences, Warsaw, Poland; 101 Department IV Fish Biology, Fisheries and Aquaculture, Leibniz Institute of Freshwater Ecology and Inland Fisheries, Berlin, Germany; 102 University of Debrecen, Centre for Agricultural Genomics and Biotechnology, Debrecen, Hungary; 103 Laboratory of Food Systems Biotechnology, Institute of Food, Nutrition, and Health, ETH Zurich, Zurich, Switzerland; 104 Sorbonne Université, CNRS, Biologie Intégrative des Organismes Marins (BIOM), Banyuls-sur-Mer, France; 105 MHNC-UP, Natural History and Science Museum of the University of Porto, Porto, Portugal; 106 Department of Agricultural Sciences, University of Naples Federico II, Portici, Italy; 107 Hellenic Centre for Marine Research (HCMR), Institute of Marine Biology, Biotechnology and Aquaculture (IMBBC), Heraklion, Crete, Greece; 108 Universite Paris Saclay, INRAE, URGI, Versailles, France; 109 Department of Genetics, University of Cambridge, Cambridge, UK; 110 Wellcome Sanger Institute, Cambridge, UK; 111 Departamento de Biologia, Faculdade de Ciencias da Universidade do Porto, Porto, Portugal; 112 Interuniversity Center for Studies on Bioinspired Agro Environmental Technology, University of Naples Federico II, Naples, Italy; 113 MARE Marine and Environmental Sciences Centre, ARNET Aquatic Research Network, Lisboa, Portugal; 114 CIBIO, Centro de Investigação em Biodiversidade e Recursos Genéticos, InBIO Laboratório Associado, Pólo dos Açores; Faculdade de Ciências e Tecnologia, Universidade dos Açores, Ponta Delgada, Portugal; 115 UNESCO, Chair Land Within Sea Biodiversity & Sustainability in Atlantic Islands, Portugal; 116 Department of Zoology, Faculty of Science, Charles University, Prague, Czech Republic; 117 Next Generation Sequencing Platform, University of Bern, Bern, Switzerland; 118 Andorra Research and Innovation, Sant Julià de Lòria, Andorra; 119 Marine Animal Ecology Group, Wageningen University and Research, Wageningen, The Netherlands; 120 Science for Life Laboratory, Department of Biochemistry and Biophysics, Stockholm University, Solna, Sweden; 121 Department of Biology and Biotechnology, University of Pavia, Pavia, Italy; 122 National Biodiversity Future Center, Palermo, Italy; 123 Centre for Ecological and Evolutionary Synthesis, University of Oslo, Oslo, Norway; 124 University of New Brunswick Saint John, Saint John, New Brunswick, Canada; 125 Institute for Medical Genetics and Applied Genomics, University of Tubingen, Tubingen, Germany; 126 NGS Competence Center Tubingen (NCCT), University of Tubingen, Tubingen, Germany; 127 Institute for Bioinformatics and Medical Informatics (IBMI), University of Tubingen, Tubingen, Germany; 128 Nature Research Centre, Vilnius, Lithuania; 129 Biodiversity Research Center, Academia Sinica, Taipei, Taiwan; 130 Faculty of Biology, University of Warsaw, Warsaw, Poland; 131 Institut Botànic de Barcelona, IBB (CSIC-CMCNB), Passeig del Migdia s.n., Parc de Montjüic, Barcelona, Spain; 132 University of Bari Aldo Moro, Department of Biosciences, Biotechnology and Environment; Institute of Biomembranes, Bioenergetics and Molecular Biotechnologies, Consiglio Nazionale delle Ricerche, Bari, Italy; 133 Department of Biology, School of Sciences and Engineering, University of Crete, Voutes University Campus, Irakleio, Greece; 134 Natural History Museum of Crete, School of Sciences and Engineering, University of Crete, Irakleio, Greece; 135 Universidade dos Acores, Departamento de Biologia, Ponta Delgada, Portugal; 136 Department of Biodiversity and Evolutionary Biology, Museo Nacional de Ciencias Naturales, Madrid, Spain; 137 Department of Ecology and Genetics, Uppsala University, Uppsala, Sweden; 138 Faculdade de Psicologia, Universidade de Lisboa, Lisboa, Portugal; 139 Linking Landscape, Environment, Agriculture and Food, Associated Laboratory TERRA, Instituto Superior de Agronomia, Universidade de Lisboa, Lisboa, Portugal; 140 Portugal Centre for Ecology, Evolution and Environmental Changes, Lisbon, Portugal; 141 Naturhistorisches Museum Bern, Bern, Switzerland; 142 Departamento de Biología de la Conservación y Cambio Global, Estación Biológica de Doñana (EBD), CSIC, Sevilla, Spain; 143 CIBER of Epidemiology and Public Health, Granada, Spain; 144 Museu Nacional de História Natural e da Ciência, Lisboa, Portugal; 145 Departamento de Biologia Vegetal, Faculdade de Ciências, Universidade de Lisboa, Lisboa, Portugal; 146 Departamento de Biologia Animal, Faculdade de Ciências da Universidade de Lisboa, Lisboa, Portugal; 147 Department of Agricultural and Environmental Sciences Production, Landscape, Agroenergy, University of Milan, Milan, Italy; 148 Department of Genetics and Genomics, Institute of Genetics and Biotechnology, Hungarian University of Agriculture and Life Sciences, Godollo, Hungary; 149 Section for Ecology and Evolution, Department of Biology, University of Copenhagen, Copenhagen, Denmark; 150 Natural History Museum, University of Oslo, Blindern, Oslo Norway; 151 Division of Evolutionary Ecology, Institute of Ecology and Evolution, University of Bern, Bern, Switzerland; 152 University of Eastern Finland, Kuopio, Finland; 153 Institute for Nuclear Research of the NAS of Ukraine, Kyiv, Ukraine; 154 Ecology, Evolution and Conservation Biology, Department of Biology, KU Leuven, Leuven, Belgium; 155 Department of Animal Ecology, Netherlands Institute of Ecology, Wageningen, The Netherlands; 156 Conservation Biology Research Group, Department of Biology, University of Malta, Msida, Malta; 157 Forest Ecology Unit, Research and Innovation Centre-Fondazione Edmund Mach, San Michele All’Adige, Italy; 158 ERISA Escola Superior de Saúde Ribeiro Sanches, IPLUSO, Lisboa, Portugal; 159 Institute for Sustainable Plant Protection, National Research Council, Sesto Fiorentino, Italy; 160 Department of Agricultural and Environmental Sciences, University of Milan via Giovanni Celoria 2, Milan, Italy; 161 Laboratory of Biodiversity and Evolutionary Genomics, KU Leuven, Leuven, Belgium; 162 Department of Zoology, Hungarian Natural History Museum, Budapest, Hungary

**Keywords:** Scientific community, Eukaryote, Genome, Genomics, Sequencing

Correction to: *npj Biodiversity* 10.1038/s44185-024-00054-6, published online 17 September 2024

In the orginial version of this Article, the wrong affiliation was given for Maria Angela Diroma. It should be Department of Biology, University of Florence, Sesto Fiorentino, Italy. In addition, her contributions were also incorrect, they should have been “M.A.D. drafted a supplementary figure/box and reviewed the draft manuscript”. The HTML and PDF versions of the Article have been corrected.

