# Supplementary material for: The European Reference Genome Atlas: piloting a decentralised approach to equitable biodiversity genomics
Source: NPJ Biodivers. 2024 Sep 17;3:28. doi: 10.1038/s44185-024-00054-6 (PMC11408602; doi:10.1038/s44185-024-00054-6)
Supplement: Supplementary file 2 — SupplementaryInformation [file 44185_2024_54_MOESM2_ESM.docx]

## **Supplementary Tables**

**Supplementary Table 1**

**Title: ERGA Sequencing Centre Partners, Biobanks and Museums Collection and Locations**

| **Sequencing Centres** | **Location** |
| --- | --- |
| [GIGA-Genomics University Liege](https://www.gigagenomics.uliege.be/cms/c_4346197/en/gigagenomics-about-us) | Belgium |
| VIB-University of Antwerp | Belgium |
| Université libre de Bruxelles (ULB) | Belgium |
| University of Copenhagen | Denmark |
| [Genoscope - Centre National de Séquençage](https://www.cea.fr/drf/ifrancoisjacob/Pages/Departements/Genoscope.aspx) | France |
| [West German Genome Center](https://wggc.de/) | Germany |
| Dresden Max Planck Institute of Molecular Cell Biology and Genetics (MPI | Germany |
| [NCCT (NGS Competence Center Tübingen)](https://portal.qbic.uni-tuebingen.de/portal/web/ncct/our-center) | Germany |
| Hungarian Centre for Genomics and Bioinformatics, University of Pécs | Hungary |
| University of Bari and Consiglio Nazionale delle Ricerche | Italy |
| University of Florence | Italy |
| Marine Animal Ecology / Animal Breeding and Genomics (Wageningen University) | Netherlands |
| Norwegian Sequencing center | Norway |
| [Centro Nacional de Análisis Genómico (CNAG)](https://www.cnag.crg.eu/) | Spain |
| [SciLifeLab](https://www.scilifelab.se/) | Sweden |
| [University of Bern, Next Generation Sequencing (NGS) Platform](https://www.ngs.unibe.ch/) | Switzerland |
| Functional Genomic Centre  Zurich | Switzerland |
| [Genomic Technologies Facility Lausanne](https://wp.unil.ch/gtf/) | Switzerland |
| [Wellcome Sanger Institute](https://www.sanger.ac.uk/) | UK |
| [Earlham Institute](https://www.earlham.ac.uk/) | UK |

**Supplementary Table 2: Roles and Responsibilities of Genome Team Members**

| **Team Member** | **Role** |
| --- | --- |
| Principal Investigator | Each genome has a designated Principal Investigator (PI) who is responsible to ensure the coordination of the project. If a sample ambassador specifically requests to lead the species genome analysis, this person will become the PI for this species’ genome. Participants covering the costs for the genomes can also request to be PI or co-PIs, in which case the sample ambassador must be in agreement. In case the participants that are covering sequencing costs are not based in the same country as the sample origin, council members from the country of origin must agree to the request. Other participants can also request to become PI or co-PIs. The sample ambassador, in agreement with other potential co-PIs, decides on the request. Co-PIs appoint a coordinator among them responsible for the project, along with organizing the handling of the samples, to ensure that proper documentation meeting the Nagoya Protocol and local regulations is provided. |
| Sample collector and/or sample provider | Responsible for the ethical and legal collection of samples for ERGA and compliance with ERGA’s ‘Sample Collection Code of Best Practices’ and ‘Data Sharing and Management Policy’. |
| Taxonomist and/or ex-situ sample manager | Individual(s) responsible for ensuring the taxonomic validity of the sample obtained and its deposition into an appropriate biobank or collection understanding that the PUID associated with both will be disclosed in the ERGA metadata manifest. |
| Sample ambassador | The sample ambassador coordinates and organizes all of the samples, permits, barcoding, and storage components of the project, up to and including the shipment to laboratory and storage of vouchers. The individual (s) should be based in the country of origin or have an established research project in the area of sampling that justifies the involvement in genome establishment for species from another country. If not based in the country, s/he must provide proof of compliance with CBD Nagoya protocol as well as permits for in-country sampling, sample import/export and handling. The sample ambassador can also act as the sample provider. |
| Wet-lab processor | Facilitates all wet lab components of the project including HMW DNA extraction, library preps, sequencing. This is a hands-  on researcher(s), or group, or could even be a facility PI. |
| Genome assembly manager | The individual(s) responsible overseeing the development, optimization, and correction of the genome assembly. Role also ensures appropriate computational resources are available. |
| Genome assembly and/or curation generator | The individual(s) responsible for the generation of the genome assembly (hands-on role). |
| Genome annotation and/or genome analysis generator | The individual(s) responsible for the generation of the gene annotation and data analysis (hands-on role). |

**Supplementary Table 3**

**Title: OmniC sequencing statistics from ERGA Sequencing Hub. Estimated genome sizes for *C. barii* and *T. fluviatilis* were obtained from** [**Genomes on a Tree (GoaT)**](https://goat.genomehubs.org/)**.**

|  | **Species** | **Tissue** | **% GC** | **Average read length (bp)** | **Read pairs (millions)** | **Unique read pairs (millions)** | **Genome size (Gbp)** | **Coverage** |
| --- | --- | --- | --- | --- | --- | --- | --- | --- |
| ***1*** | ***Botryllus schlosseri***  (Golden Star Tunicate) | whole specimen | **42%** | **150** | **282.6** | **155.1** | **0.70** | **66** |
| ***2*** | ***Cryptocephalus barii*** | whole specimen | **43%** | **150** | **305.3** | **180.6** | **0.50** | **108** |
| ***3*** | ***Hottonia palustris***  (water violet) | whole specimen | **37%** | **151** | **129.6** | **108.5** | **0.86** | **38** |
| ***4*** | ***Knipowitschia panizzae***  (Adriatic dwarf goby) | gills | **45%** | **150** | **104.4** | **88.9** | **0.87** | **31** |
| ***5*** | ***Lepus granatensis***  (Granada hare) | kidney | **45%** | **150** | **578.1** | **443.4** | **2.65** | **50** |
| ***6*** | ***Mullus barbatus***  (red mullet) | liver | **47%** | **150** | **32.8** | **30.4** | **0.54** | **17** |
| ***7*** | ***Oenanthe leucura***  (Black wheatear) | blood | **44%** | **150** | **339.3** | **252.4** | **1.36** | **56** |
| ***8*** | ***Parnassius mnemosyne***  (Clouded Apollo) | whole specimen | **40%** | **151** | **29.1** | **25.3** | **1.46** | **5** |
| ***9*** | ***Salvelinus alpinus***  (Arctic char) | liver | **45%** | **150** | **386.4** | **275.5** | **2.73** | **30** |
| ***10*** | ***Theodoxus fluviatilis***  (river nerite) | whole specimen | **48%** | **150** | **321.2** | **177.1** | **0.86** | **62** |
| ***11*** | ***Tripterygion tripteronotum***  (red-black triplefin) | brain | **44%** | **149** | **236.2** | **204.3** | **0.77** | **79** |

**Supplementary Table 4: RNA-seq statistics from ERGA Sequencing Hub**

| **Species** | **No. of libraries** | **Tissues** | **% Duplications** | **% GC** | **Average read length (bp)** | **Read pairs (millions)** |
| --- | --- | --- | --- | --- | --- | --- |
| ***Asellus aquaticus***  (water hoglouse) | 1 | whole specimen | **81%** | **39%** | **141** | **38** |
| ***Acanthodactylus schreiberi***  (Schreiber's fringe-fingered lizard) | 4 | brain, kidney, liver, muscle | **51%** | **47%** | **136** | **124** |
| ***Andrena vaga***  (grey-backed mining bee) | 1 | whole specimen | **63%** | **46%** | **132** | **119** |
| ***Bufotes viridis***  (European green toad) | 7 | heart, kidney, liver, lung, muscle, spleen, skin | **55%** | **46%** | **136** | **217** |
| ***Corema album***  (Portuguese crowberry) | 1 | leaf | **77%** | **52%** | **88** | **4** |
| ***Cryptocephalus barii*** | 2 | whole specimens | **78%** | **43%** | **132** | **277** |
| ***Hottonia palustris***  (water violet) | 4 | old buds, young buds, leaves, flowers/petals | **65%** | **48%** | **138** | **94** |
| ***Laurus azorica***  (the Azores laurel) | 1 | leaf | **60%** | **52%** | **121** | **6** |
| ***Lepus granatensis***  (Granada hare) | 5 | kidney, liver, lung, spleen, testes | **50%** | **54%** | **136** | **179** |
| ***Mullus barbatus***  (red mullet) | 4 | fin, gonad, kidney, muscle | **60%** | **52%** | **126** | **262** |
| ***Nepa anophthalma***  (Stygobiotic Waterscorpion) | 1 | whole specimen | **81%** | **36%** | **140** | **53** |
| ***Oenanthe leucura***  (Black wheatear) | 1 | blood | **77%** | **54%** | **128** | **93** |
| ***Palingenia longicauda***  (Tisza mayfly) | 2 | larvae | **90%** | **39%** | **140** | **75** |
| ***Parnassius mnemosyne***  (Clouded Apollo) | 1 | whole specimen | **76%** | **42%** | **138** | **94** |
| ***Spinachia spinachia***  **(**[Fifteen-spined stickleback](https://www.marlin.ac.uk/species/detail/2081)**)** | 4 | pelvic fin and muscle, internal organs, brain and eyes, gills | **51%** | **51%** | **133** | **171** |
| ***Stylops ater*** | 2 | whole specimens | **70%** | **39%** | **139** | **115** |
| ***Theodoxus fluviatilis***  (river nerite) | 1 | foot | **74%** | **45%** | **138** | **44** |
| ***Trechus terceiranus*** | 1 | whole specimen | **73%** | **41%** | **142** | **52** |
| ***Zostera noltei***  (dwarf eelgrass) | 1 | leaf | **86%** | **49%** | **135** | **74** |
| ***Zygaena transalpina***  (Transalpine Burnet Moth) | 1 | whole specimen | **62%** | **43%** | **144** | **66** |

## **Supplementary Notes**

**Supplementary Note 1**

**Title: Navigating Nagoya Compliance**

Ten genome teams across eight countries and regions (Malta, Azores, Croatia, France, Greece,, Hungary, Portugal, and Slovakia) had to obtain a Nagoya permit prior to collecting samples for the project. For all countries the process for obtaining a permit in a relatively short period of time, averaging two months, was centred on an initial engagement with the Access and Benefit Sharing (ABS) National Focal Point (NFP) or Competent National Authority (CNA). Both NFPs and CNAs offered initial guidelines for the specific national procedures. Some countries have online portals to streamline the Nagoya permitting process e.g, France (https://www.ecologie.gouv.fr/acces-et-partage-des-avantages-decoulant-lutilisation-des-ressources-genetiques-et-des-connaissances), Azores (<https://servicos-sraa.azores.gov.pt/doit/servicos.asp?id_dep=3&id_form=18>) and Malta (<https://www.servizz.gov.mt/en/Pages/Environment_-Energy_-Agriculture-and-Fisheries/Agriculture/Agriculture/WEB05310/default.aspx>). Other countries had easily accessible downloadable application templates, whilst others coordinated permitting through email engagements with the National Focal Point (Azores) being asked to provide information regarding: sampling, uses, intent to transfer and confirming they would cooperate with any knowledge transfer or benefit sharing obligations.

Most sample providers declared non-commercial use (research purpose) for the sample collection purposes. The terms and conditions of the IRCCs obtained were diverse and ranged in complexity. For Azores, the IRCC laid out provisions for sampling, reporting, benefit-sharing and third-party transfers. In terms of benefit-sharing, the Azorean team ensures the disclosure of the IRCC permit ID in all scientific publications, a key form of non-monetary benefit sharing[^1^](https://paperpile.com/c/HBikTh/JJYSO). For France, just sampling details were required and the initial permit has to be updated if a change in utilisation occurs (further biochemical analyses, commercial uses etc…). For Croatia, a short report on the samples collected after the duration of the permit was requested along with the collection methodology and number of specimens collected.

For Malta, terms and conditions were more comprehensive including: notification if a change in contract terms was required; compliance with local authorities; respectful use of Traditional Knowledge if relevant; a summary report on project conclusion; copy of all associated research publications (must be made freely available and attribute the sample provider country); annual progress reports; documentation storage for 20 months after project completion; inform local authorities when data becomes publicly available; and compliance with Europe’s Due Diligence requirements through ‘DECLARE’. For Hungary, the initial permit had been previously acquired, outlining the terms for collection of *Vipera ursinii rakosiensis* samples to facilitate a genetic screening for a reintroduction program of this endangered species. For the purposes of ERGA and sample sequencing in the UK, the sample ambassador had to obtain a new permit in order to send specimens outside of the EU. The permitting process was free of cost for most teams apart from Hungary (20,000 HUF). Four genome teams also required CITES permits.

**Supplementary Note 2**

**Title: Sample Collection and DNA extraction barriers**

Criteria for inclusion were developed for ERGA to prioritise species where genomic data would have immediate potential impacts e.g., endangered species. However, to ensure feasibility, more straightforward species were also prioritised, e.g., haploid, <~1Gb genome size etc.. In practice however, acquiring suitable samples presented challenges for some species, for instance in the case of the threatened Siberian flying squirrel (*Pteromys volans*) and the golden jackal (*Canis aureus*). Due to the threatened status of the flying squirrel, only pre-existing samples could be obtained. These initial samples belonged to found-dead animal, however during DNA extraction the samples yielded insufficient levels of high molecular weight DNA (HMW-DNA). After this, a sample from an ear biopsy from a live animal was obtained, but again yielded inadequate HMW-DNA. Similarly for the jackal, two samples taken from dead animals and stored at -80 had inadequate HMW-DNA volumes. The Rhône Streber, one of the rarest and most strongly threatened fish species of Europe, also highlighted the complexity of sampling endangered species. Over the past decade, this species has declined sharply both in Switzerland and France and lately has fallen below the limit of detection in Switzerland. Obtaining samples from the wild population was not permitted however, fortunately a single individual was provided for the purposes of the ERGA from an international ex-situ breeding program at Aquatis Aquarium Vivarium Lausanne.

Interestingly, in a few cases (*Lepus timidus* & *Lepus Europaeus)*, reference genomes were successfully produced from HMW-DNA extracted from fibroblast cell lines. This technique did not require invasive sampling or large tissue volumes. Utilizing ex-vivo cell lines could provide a solution for producing reference genomes for a wide range of species where obtaining fresh, flash frozen samples may be a challenge, particularly endangered or protected species, as it results in minimal harm to the individual or population.

Intentionally prioritising species with smaller genome sizes resulted in additional challenges. For instance, for the reference genome for *Stylops ater* (body weight =2mg) [^2^](https://paperpile.com/c/HBikTh/XWkK), DNA from a single individual was ideal and so an amplification step was undertaken. This resulted in the successful production of long reads but failure of HiC data, and so the assembly produced failed to meet the EBP metric as it could not be adequately scaffolded and curated. *Gyrodactylus teuchis*, a monogenean species, was prioritised for inclusion as it would be the first species within the entire Monogenea class of parasitic flatworms to have a reference genome produced. Due to its small size, a single individual yielded only 1ng of DNA and the pooling of worms would introduce unwanted sequence variation into the reference genome. The challenge of DNA extraction and library preparation from single worms was tackled using specific low-input protocols by the team of the Darwin Tree of Life initiative. *G.teuchis* is an obligate parasitic species living and feeding on the skin of its host species, making the extraction of DNA, without contamination from the host species, essentially impossible. Further, the genome of the host (in this case *Salmo trutta*) is about two orders of magnitude larger than the parasites, so even minute contamination with host cells will lead to considerable contamination at the read level. Such reads will be removed rigorously using the reference genome of the host[^3^](https://paperpile.com/c/HBikTh/JJVK) (), as well as in the assembly process through iteratively assessing assemblies and filtering based on aggregate properties, such as coverage and GC content[^4^](https://paperpile.com/c/HBikTh/LAKd). For *Stylops ater* this was solved by sampling the free-living stage of adult males and not females that never leave the host body[^5^](https://paperpile.com/c/HBikTh/Evpc)*. Cladonia norvegica*, a lichen symbiosis, also yielded low volumes of HMW-DNA, with contamination (at least one fungus, one alga and many bacteria) being problematic for successful DNA extraction. Although sequencing has not yet been completed for this organism, it is highly likely to also contain mite DNA, a regular inhabitant of this symbiosis. Even with tissue free of extracellular contamination, intracellular symbiotic or parasitic bacteria can pose a challenge. An estimated 20% of all insects are infected with Wolbachia[^6,7^](https://paperpile.com/c/HBikTh/5Evp+Y2If). Genomes of three different strains of Wolbachia, two complete and one incomplete, could be filtered out and assembled from the long-read data of *Stylops ater*.

Such cases highlight the challenges faced when producing high quality reference genomes from endangered and threatened species as the production of genomes that meet the EBP metrics require large quantities of HMW-DNA that can more likely be obtained by freshly collected and flash-frozen samples. It also showcases the need for adequately training biodiversity researchers new to the field of reference genome production, prior to sample collection, to understand the importance of sampling methodology, specifically in terms of tissue type, tissue quality, preservation method and storage. For 48% of the teams participating, it was the first experience in producing high quality reference genomes and therefore, many were inexperienced in the community accepted best practices for sampling. Without sufficient training prior to sample collection, the likelihood of samples of suboptimal quality being sent for sequencing is much greater, resulting in the wasting of time-, financial- and personnel resources.

**Supplementary Note 3**

**Title: Swiss Hi-C Protocol Challenges**

Attempts at generating sufficient Hi-C data for the two bees (*Andrena humilis* and *Osmia cornuta*), two beetles (*Carabus intricatus* and *C. granulatus*), and the mayfly (*Epeorus assimilis*) were ultimately unsuccessful. The first trial was performed using *A. humilis* thorax tissue following the ProximoTM Hi-C Kit (Animal) Protocol v4.0 from Phase Genomics. This yielded 27 Gbp from 90M reads, however, contamination checks revealed 75% of reads mapping to *Pseudomonas* bacteria leaving insufficient read coverage for scaffolding. For the second set using the same protocol, head tissues were used for the bees and the mayfly while leg tissue was used for the beetles. Sequencing yielded variable read counts of 290M *A. humilis*, 23M *O. cornuta*, 212M *C. intricatus*, 275M *C. granulatus*, 100M *E. assimilis*, however after deduplication there remained only 14%, 5%, 29%, 24%, and 6% of reads, respectively. The high levels of duplicates observed resulted from performing more than the maximum recommended number of PCR cycles during amplification steps in an attempt to increase overall yield. The low numbers of unique and mappable reads meant that the read coverage obtained for each of the five species was not sufficient to use for scaffolding the primary assemblies. Resampling is now underway (*E. assimilis* and *C. granulatus* collected, others ongoing) to collect new individuals from which to obtain samples.

**Supplementary Note 4**

**Title: Situating ERGA inside the global biodiversity community**

As part of its commitment to biodiversity research, ERGA is keenly aware of the importance of preserving biodiversity hotspots and unique ecosystems, and strives to be involved in conservation campaigns to protect them. An example of such a campaign is the one to protect Ayyalon Cave in Israel, a unique isolated ecosystem based solely on chemoautotrophic food production by sulfur-oxidising microorganisms[^8^](https://paperpile.com/c/HBikTh/HmtH). The cave was discovered inside an active quarry in central Israel in 2006. It has probably been isolated from the surface for as long as six million years [^9^](https://paperpile.com/c/HBikTh/tA2D). Nearly all of the species found in the cave were new to science and are endemic to this ecosystem. The cave and its specialised fauna first came to ERGA’s attention when a suggestion was advanced to sequence the genomes of some of its unique species as part of the pilot project. Shortly afterwards it emerged that the very existence of the Ayyalon Cave ecosystem was under threat due to a planned project to use the quarry in which the cave resides as an overflow reservoir for flood management. Allowing flood waters into the quarry would almost definitely inundate the cave, disrupting its unique food web, and would lead to the extinction of its endemic fauna.

A group of Israeli scientists and conservationists rapidly mobilised to oppose this plan. They organised a public campaign with a series of online petitions on various platforms, wrote professional letters to relevant government agencies and decision makers, and appealed to the international scientific community to provide letters of support for the protection of Ayyalon Cave [^10^](https://paperpile.com/c/HBikTh/d0HK). ERGA was one of the international organisations and societies providing letters of support. Ultimately, the public campaign and international support were successful[^10^](https://paperpile.com/c/HBikTh/d0HK), the flood management plan was modified so as to not include Ayyalon quarry, and the cave was saved. This story highlights the role that biodiversity genomics initiatives can play not only in the effort to document biodiversity but also in the never-ending struggle to preserve it.

**Supplementary Note 5**

**Title: Accessibility of Cold Chain Shipment**

Sample quality and DNA integrity are essential for the extraction of HMW DNA, which in turn is essential for the production of complete reference genomes that rely on long-read data. To this end, sample collection, preservation and storage are key to the successful production of high-quality reference genomes. To increase the likelihood of success, and in accordance with community-accepted best practices, ERGA endorsed all samples to be ethically and legally sourced, immediately flash frozen, and stored at -80ºC.

For sustaining sample integrity during shipment to ERGA-Pilot associated sequencing facilities, shipment on a continuous cold chain using dry ice was preferred. In 2022, there was a global shortage of dry ice due to the rising cost of gas and other factors that greatly impacted ERGA-Pilot causing weeks of delays in shipping for some teams. Interestingly, 41% of teams (n=93) experienced a challenge during shipment, and 43% required additional samples to be sent. Most reported insufficient sample quality or DNA quantity as the main reason for reshipment. Several reported delays in courier shipment as the cause for sample quality degradation.

Almost 50% of teams paid between €100- €500 per shipment, and 34% < €100. However, for many teams that had less genomics experience or from a country/region that was under-resourced regarding genomics, the costs associated and the certifications required for cold chain shipping were prohibitive and made this an inaccessible option. Highlighting this issue was the sulfidic groundwater aquifer samples collected from the deep recesses of Movile Cave. Here, old 20 m deep hand-dug drinking wells were the only windows of access making sample collection challenging and requiring expertise in single rope techniques to reach the sulfidic sites. Even more challenging was shipping these samples from the cave to the sequencing centre on a cold chain, with the shipment cost estimated at €300 per sample - a prohibitive expense for the genome team. The most viable and cost-effective option for the team was to purchase a roundtrip plane ticket at €80 and travel with the live samples to the sequencing centre.

In other cases, teams experienced a reluctance from couriers to send biological materials on dry ice. Despite declaring on public-facing websites their ability to do so, when contacted their response was either negative or expressed a requirement for a shipment certificate. These required certificates take time to obtain but also have associated costs and would potentially be under-utilised by those sending only a few samples. As a result, it was arranged that it would be more time- and cost-effective to hand deliver some samples. One team travelled 900 km and met halfway with colleagues from the sequencing centre.

Moving forward, ERGA will test alternative and less costly methods for shipment e.g., DNA/RNA Shield for RNAseq samples, to increase the accessibility of the production of reference genomes to all across Europe. ERGA will also consider how to coordinate the shipment of species from a country, weighing up whether it is more time- and cost-effective to first centralise the samples within a country so that a single shipment can be conducted, or alternatively ship samples from multiple locations across the same country. Additionally, as ERGA grows and gains insights into the permitting and certification procedures necessary for shipment across European countries, it could become possible to develop shipment guidelines to support participating researchers or indeed develop partnerships with courier services with centralised ERGA accounts to streamline the process and potentially obtain discounted prices.

**Supplementary Note 6**

**Title: Experiences from ERGA Library Preparation Hubs**

Having dedicated resources (financial, infrastructural, personnel) to facilitate members from institutions, regions, or countries that are equity deserving in terms of genomics research would greatly expedite the successful utilisation of the infrastructure. For the pilot test, the ERGA Library Preparation and Sequencing Hubs stood at the front-line of tackling equity barriers, and so faced several challenges. One such challenge was obtaining samples that were of a quality and quantity that could support long-read and Hi-C data production [**Supplementary Note 2].** Many samples needed to be recollected and reshipped, e.g., *Trechus terceiranus* (icTreTerc1), a cave adapted endemic beetle from the island of Terceira (Azores, Portugal), was resampled due to sample spoilage caused by dry-ice evaporation during cold-chain shipment [see **Supplementary Note 5**]. DNA extraction and library construction from recalcitrant species also presented challenges [**Supplementary Note 2,3**]. For instance, *Palingenia longicauda* (iePalLong1) has a large cuticle-to-tissue ratio and the presence of large wings interfered with our ability to obtain suitable samples, and *Stylops ate*r (ivStyAter1), an endoparasite of the grey-backed mining bee *Andrena vaga*, failed the library preparation step multiple times due to a limited volume of starting material. Finally, for plant species RNA containing ribosomes from different organelles results in multiple RNA bands making it challenging to accurately analyse the integrity of the RNA. Additionally, for both arthropods and molluscs the 28S subunit rRNA is susceptible to a gap deletion that causes band fragmentation. This collapse appears as a single band that resembles the 18S rRNA subunit that can easily be misinterpreted as rRNA degradation with the Rin Integrity Numbers obtained being extremely low[^43^](https://paperpile.com/c/E41j9b/KKxC).

**Supplementary Figures**


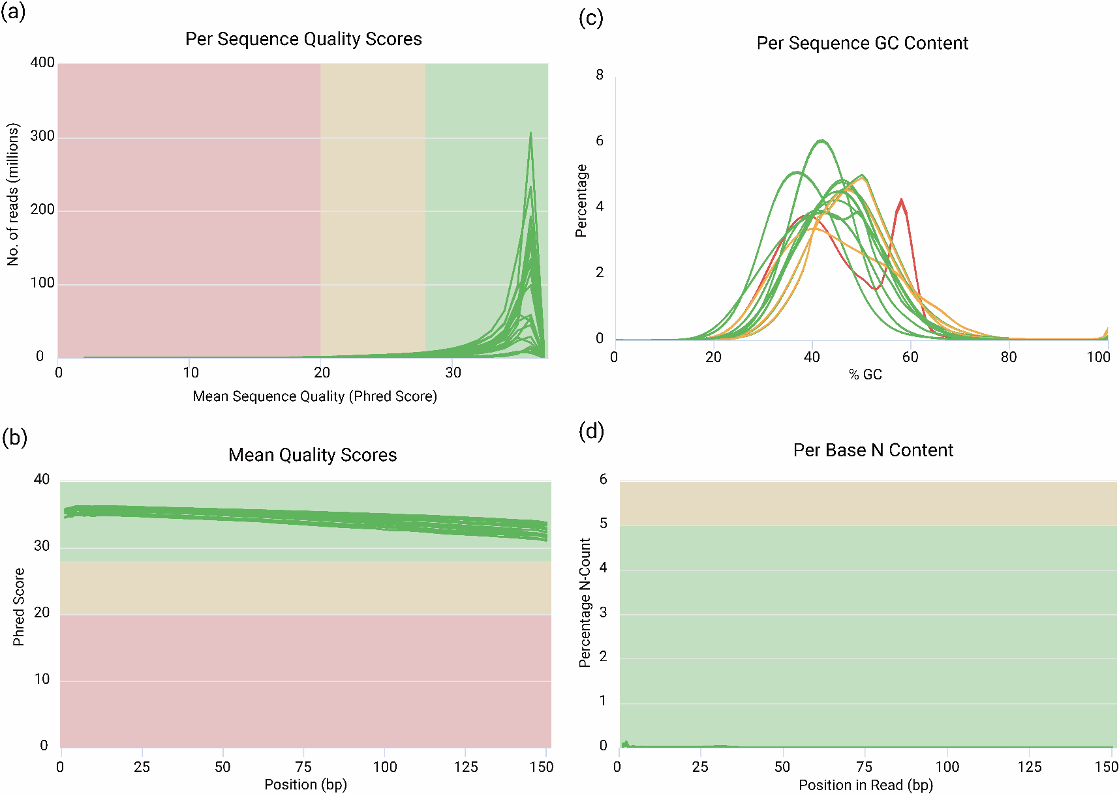


**Supplementary Figure 1:** Quality Assessment of Hi-C Sequencing Data. (*a*) Per Sequence Quality Scores: Distribution of quality scores across all sequences, with the y-axis representing the number of reads in millions. (*b*) Mean Quality Scores: The average quality score at each position in a read. (*c*) Per Sequence GC Content[HL2] : The GC content distribution across all sequences. (*d*) Per Base N Content: The proportion of N content at each base position in the reads, reflecting ambiguous base calls.


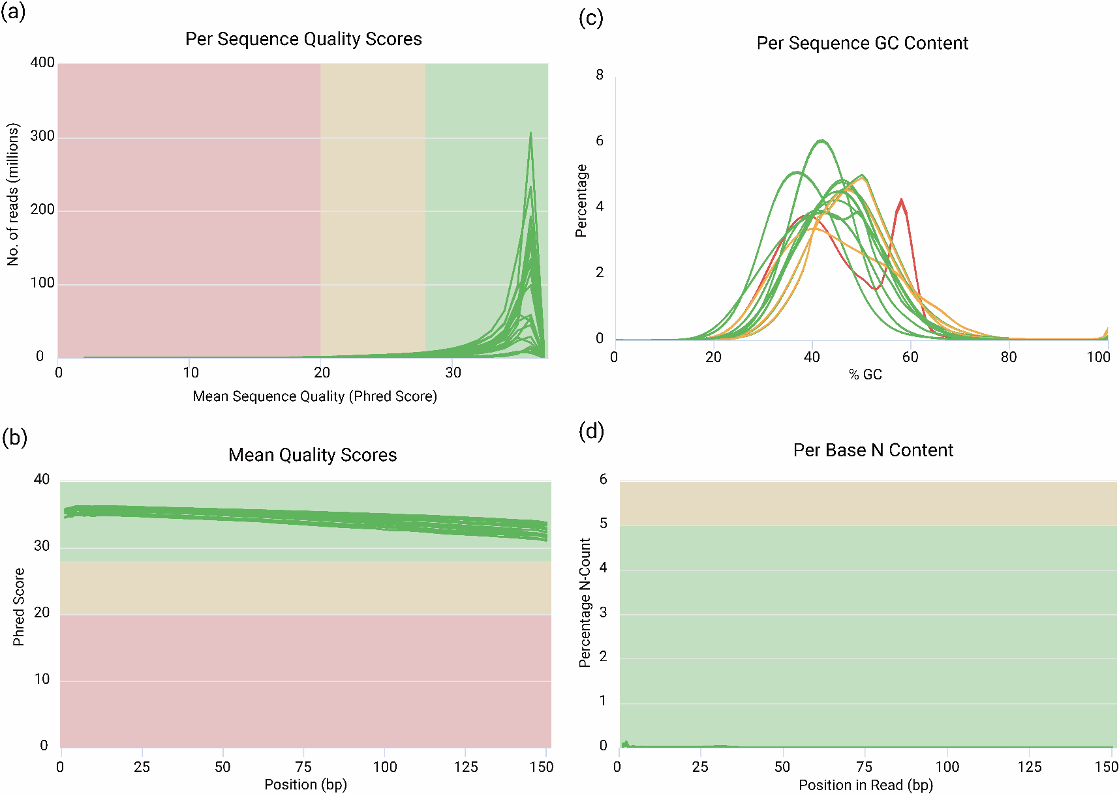


**Supplementary Figure 2:** Quality Assessment of Hi-C Sequencing Data. (*a*) Per Sequence Quality Scores: Distribution of quality scores across all sequences, with the y-axis representing the number of reads in millions. (*b*) Mean Quality Scores: The average quality score at each position in a read. (*c*) Per Sequence GC Content[HL2] : The GC content distribution across all sequences. (*d*) Per Base N Content: The proportion of N content at each base position in the reads, reflecting ambiguous base calls.


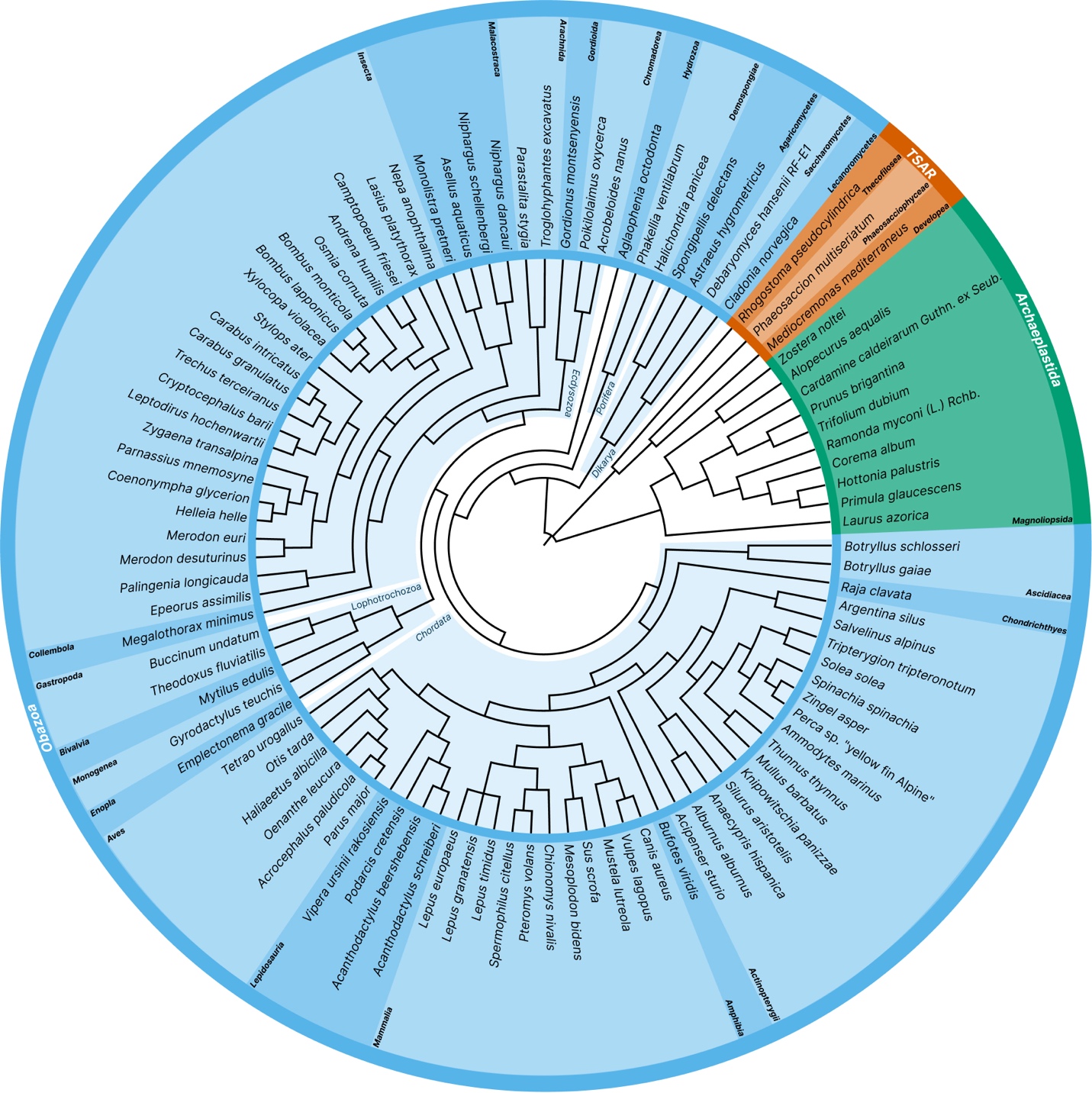


**Supplementary Figure 3:** Phylogeny of all the species participating in the ERGA Pilot Project


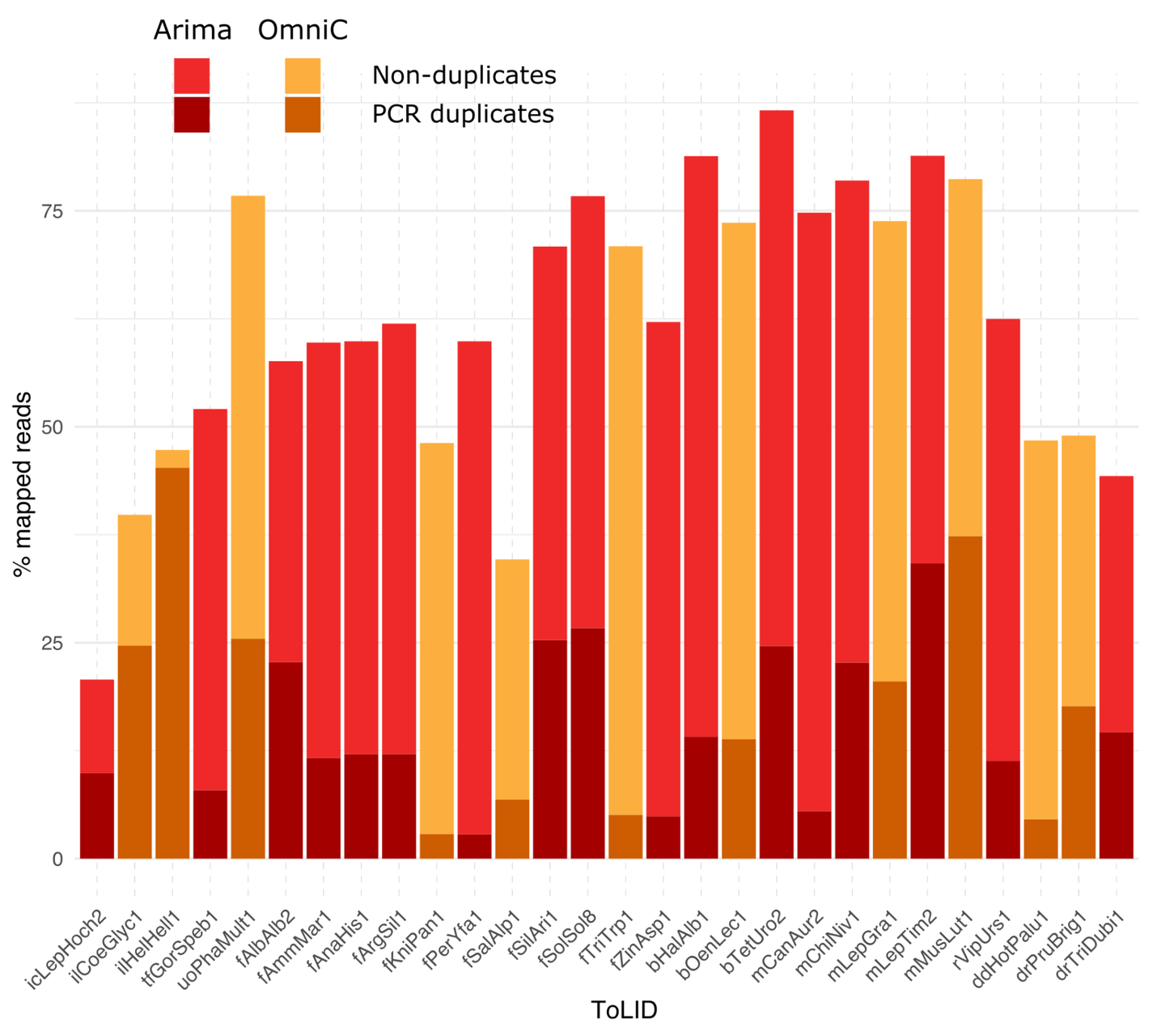


**Supplementary Figure 4:** Hi-C reads mapping metrics for completed (pre-curation and curated) assemblies according to kits used (Arima or OmniC).


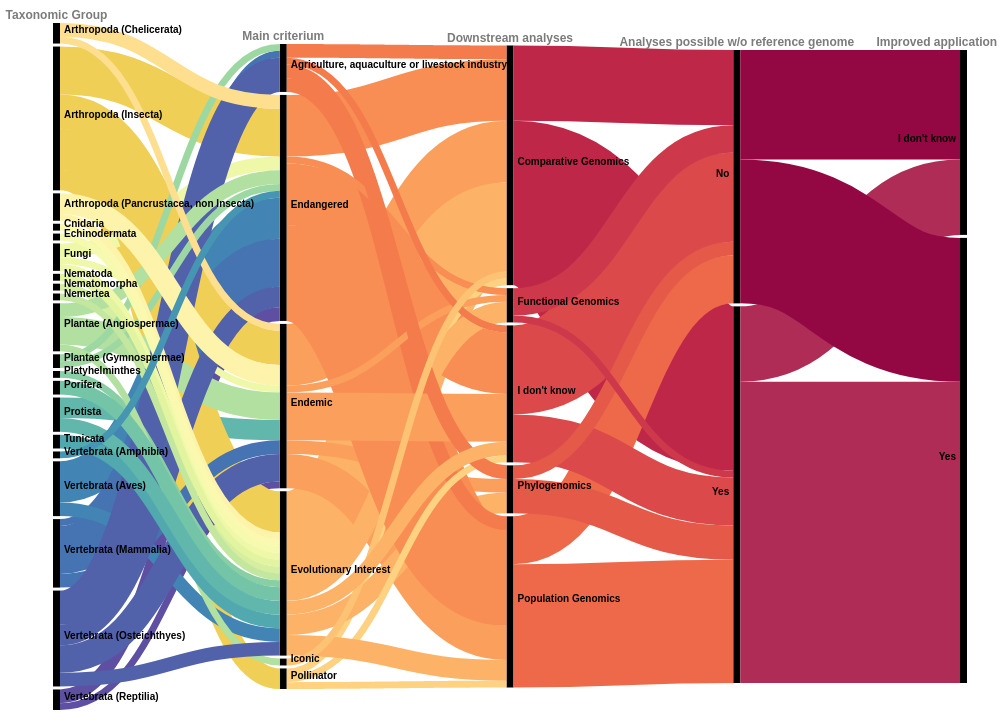


**Supplementary Figure 5:** Summary of genome team data analysis survey responses.


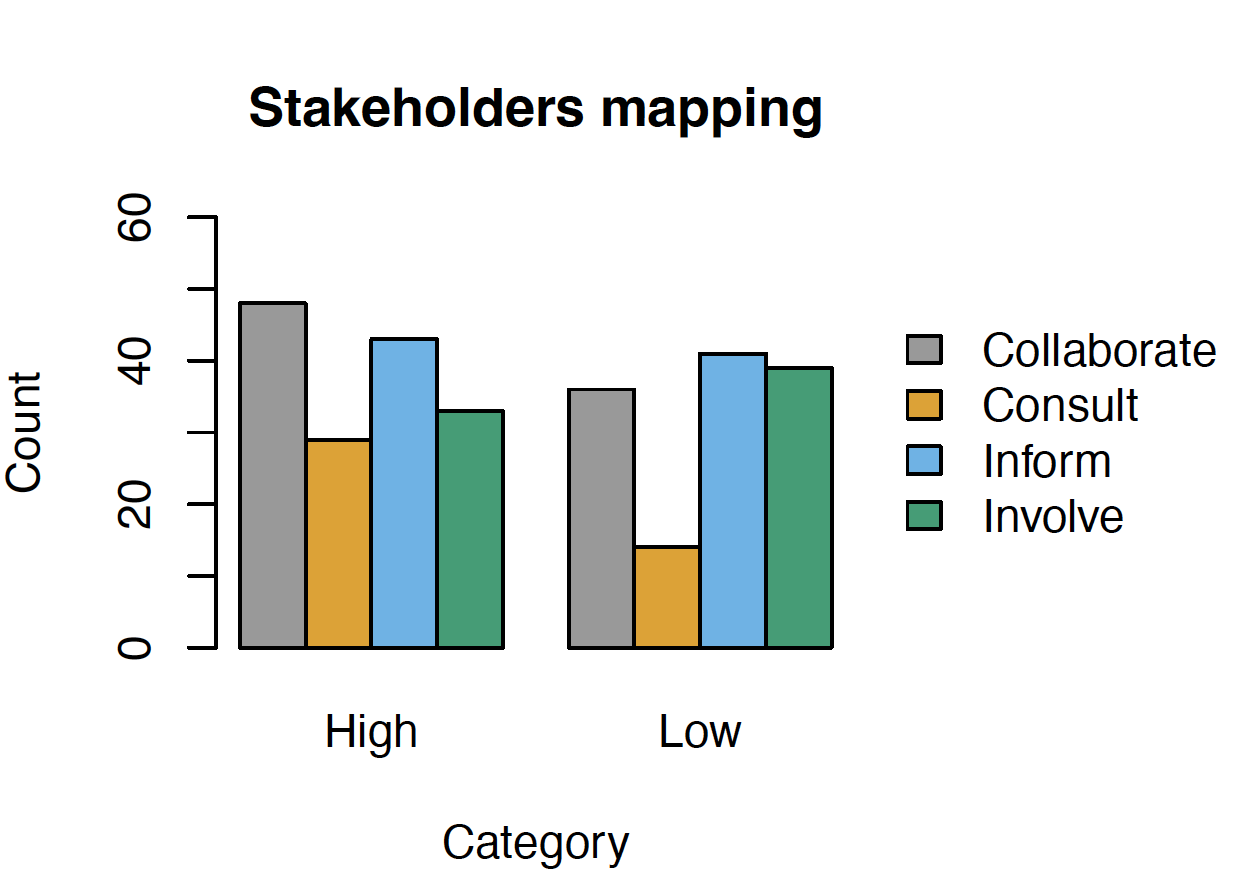


**Supplementary Figure 6:** The differences in how participants from countries with a GBARD higher than 1000 MM (High) and those with a GBARD lower than 1000 MM (Low) divided interested parties into the four categories of engagement.


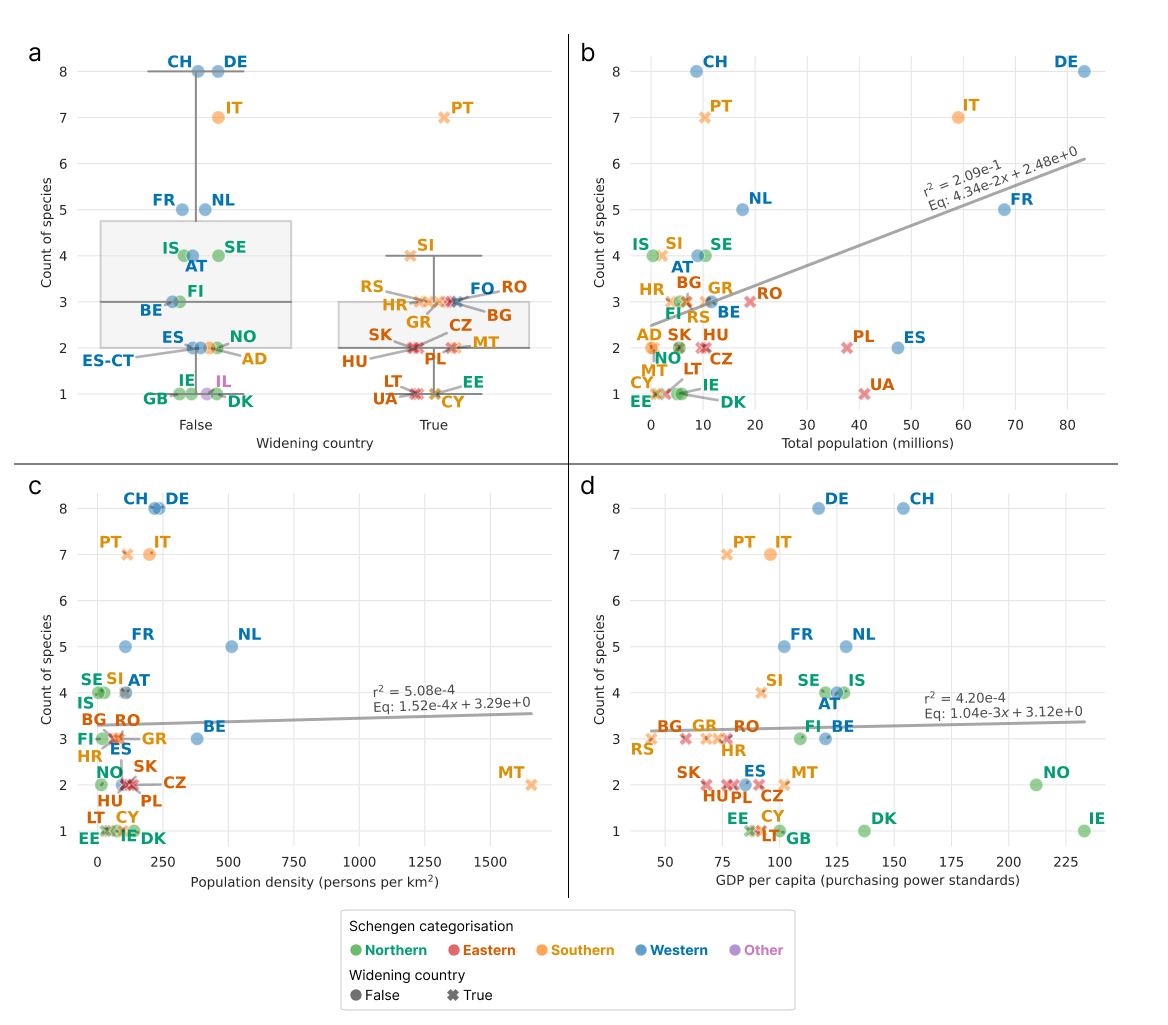


**Supplementary Figure 7:** Relationship between number of species attributed per country and country characteristics. Distribution of the number of species attributed per country: (a) Categorized as non-Widening or Widening; (b) Total population; (c) Population density; (d) Gross Domestic Product (GDP) per capita in purchasing power standards. Data points labelled with ISO country codes. Country-level data for population, population density and GDP per capita in PPS, as sourced from Eurostat (2022).

**Supplementary Glossary**

**Biodiversity genomics** - The application of genomic methods to research biodiversity.

**BUSCO** -  A bioinformatic method (Benchmarking Universal Single-Copy Orthologues) used to estimate the completeness of the coding fraction of an organism’s genome based on the proportion of (lineage specific) single copy orthologous genes that are found in a genome assembly [^52^](https://paperpile.com/c/mqhYHr/kRV57).

**INSDC** - International Nucleotide Sequence Database Collaboration (<https://www.insdc.org/>) is an initiative between the DDBJ, EMBL-EBI and NCBI  that together act as a global repository of sequence data and associated metadata, and provide tools and services that allow access to genomic resources.

**Reference** **genome**  - An accepted standard representation of an organism’s DNA sequence. High-quality reference genomes typically have high completeness (chromosome-level with few gaps in sequence), few errors, and are annotated and accessible. A reference genome serves as a tool for alignment-based analyses, such as variant calling or RNAseq, and has many other applications, for example, phylogenetics and evolutionary relationships, identification of genes and variants, functional analysis and comparative genomics. Reference genomes referred to as “drafts” are those that are under active construction and refinement, and not yet finalised through manual curation.

**Genomic resource -** A genomic resource, for the purpose of this manuscript, refers to a reference genome, genome annotation, voucher specimen, cryopreserved sample and comprehensive metadata.

**FAIR Principles** **-** A set of principles to guide appropriate management and curation of scientific data (<https://www.go-fair.org/fair-principles/>) that emphasise data accessibility and use by ensuring that data are Findable, Accessible, Interoperable, and Reusable. Due to the increasing amount of scientific data being reposited, FAIR guidelines promote a data format that is amenable to automated computational access of data by stakeholders[^64^](https://paperpile.com/c/mqhYHr/wbuRn).

**CARE** **Principles -** The CARE principles for Indigenous data governance (<https://www.gida-global.org/care>) provide a governance framework that supports the recognition of rights and interests Indigenous Peoples’ to their physical and digital data as well as their Indigenous Knowledges[^65^](https://paperpile.com/c/mqhYHr/toXby).

**Metadata** **-** A collection of data that provides contextual information about multiple characteristics of other, corresponding original data.

**Voucher**  **-** A voucher specimen is a permanently preserved object (either whole or in part, and/or physical or digital) of an identified organism (verified by a recognised expert) and which is deposited in an accessible facility or database. A voucher provides physical evidence about any specimen’s taxonomic identity[^14^](https://paperpile.com/c/mqhYHr/6tZQ0). Voucher deposition is a best practice for conducting biodiversity genomics research.

(Genome) **annotation** **-** The process of identifying the functions of different pieces of a genome. This includes genes that code for proteins and non coding features (e.g. intron-exon structure of protein coding genes, promotors, transposable elements). Typically performed using computational methods, followed by manual curation.

(Genome) **completeness** **-** An estimate of how well a reference genome represents the complete sequence of the target organism. A complete genome should equal the haploid genome size of the target, but may be defined when ‘*all chromosomes are gapless and have no runs of 10 or more ambiguous bases, there are no unplaced or unlocalized scaffolds, and all expected chromosomes are present.*’ ([https://www.ncbi.nlm.nih.gov/assembly/](https://www.ncbi.nlm.nih.gov/assembly/help/)). There are different approaches to estimate the completeness, like BUSCO, analysing K-mers, etc.

**Library** **-** DNA, cDNA, or RNA that has been prepared for NGS within (usually) a specific size range and containing adapters, which are designed to be appropriate for (a) specific sequencing platform(s).

(Genome) **assembly** **-** A genome assembly is a representation of an organism’s genome that is made using computer programs to turn (assemble) raw sequence data into longer, continuous sequences.

**PUID -** A permanent unique identifier is a unique label for an object that does not change, such as the Digital Object Identifier (DOI) attached with a scientific publication.

**ENA** **-** The European Nucleotide Archive (<https://www.ebi.ac.uk/ena>) is a global repository for sequence data and provides resources that support management and access to sequence data.

**Equity Deserving** - According to the Canadian Council (https://canadacouncil.ca/glossary/equity-seeking-groups) equity deserving groups are those individual researchers, communities, Peoples, regions or countries that have identified barriers to equal access, opportunities, and resources due to disadvantage and/or discrimination and that are actively seeking, and deserving of  social justice and reparation. The discrimination experienced could be caused by attitudinal, historic, social, and environmental barriers that could be based on a plethora of characteristics that are including (but not limited to) sex, age, ethnicity, disability, economic status, gender, gender expression, nationality, race, sexual orientation, and creed.

**COPO** **-** The Collaborative OPen Omics (COPO) platform is for researchers to publish their research assets, providing metadata annotation and deposition capability. It allows researchers to describe their datasets according to community standards and broker the submission of such data to appropriate repositories whilst tracking the resulting accessions/identifiers[^29^](https://paperpile.com/c/mqhYHr/moND8).

**Open data** **-** Open data are freely accessible and unrestricted data that can be accessed, used, reused and shared with third parties for any purpose.

**HSM** **-** Hierarchical Storage Management is both a data management and data storage technique which transparently manages the movement of data between the different layers of a tiered storage based on file size thresholds, usage and I/O pressure. Usually, a tiered storage is composed of one or more layers of disk arrays, ordered by capacity, latency, redundancy and storage cost. A slow but economically effective archival layer is at the bottom, composed of magnetic tape libraries and automated tape robots, with the highest capacity and latency. The movement between layers is automatically triggered.

**ONT** **-** Oxford Nanopore Technologies (ONT; <https://nanoporetech.com/>) is a next generation sequencing technology whereby sequence data are generated from the changes in current that occur as single-stranded DNA or RNA molecules pass through nanoscale protein pores (nanopores). ONT provides long read data (up to several megabases) that facilitate genome assembly[^66,67^](https://paperpile.com/c/mqhYHr/G8rTD+giifP).

**PacBio** **-** Pacific Biosciences (PacBio; <https://www.pacb.com/>) is a single-molecule, real time (SMRT) next generation sequencing technology in which sequence data are generated by fluorescent light emission that occurs when a DNA polymerase adds nucleotides. PacBio produces long read data (tens of kilobases) that facilitate genome assembly.

**HiFi reads** **-** HiFi (High Fidelity) PacBio reads are produced by taking multiple sequences of the same molecule to provide a consensus sequence that is usually 12-20kbp long and has a low error rate (>99.9 % consensus accuracy)[^68^](https://paperpile.com/c/mqhYHr/MQGaR).

**Hi-C** **-** Sequencing-based method used to study three-dimensional interactions among chromatin regions by measuring the frequency of contact between pairs of loci. Since contact frequency is related to the distance between a pair of loci, Hi-C linking information is used to help with scaffolding stages during a genome assembly process.

**Hi-C map / graph production** **-** The occurrence and frequency of Hi-C contacts are analysed and used in assembly scaffolding. They are typically visualised in Hi-C 2D heatmaps with the full genome sequence on the X and Y axis and a markup for each observed contact.

**Omni-C** **-** Modified version of Hi-C that uses a sequence-independent endonuclease during its protocol to produce more even sequence coverage increasing overall resolution.

**RNA-Seq** **-** RNA-Seq is a technique that determines the complete or partial RNA sequence using NGS. The RNA expression profiles vary in different tissues of the same organism and can be influenced by physiopathological circumstances. RNA-Seq data facilitate genome assembly by providing empirical evidence for annotation of transcribed regions[^69^](https://paperpile.com/c/mqhYHr/Vdp2L).

**IsoSeq** **-** This is a sequencing protocol developed by PacBio that aims to sequence full-length transcripts using the accurate, long read capabilities of PacBio HiFi technology. IsoSeq data facilitate analysis of transcriptomes and genome annotation by identifying full-length isoforms of transcripts.

**Haplotype** **-** A haplotype refers to the collection of genetic material within an organism that is inherited together. Haplotype may be used to describe a few loci or any number of chromosomes (a chromosome-scale haplotype).

**K-mer** **-** A K-mer is a DNA sequence of length k; for example, the sequence AGCT contains the 3-mers (K-mers of length 3) AGC and GCT.

**Transcriptome** **-** A transcriptome is a set of aligned RNAseq reads representing RNA collected from a sample or collection of samples. This includes both protein-coding and non-coding transcripts. For the ERGA Pilot Project, poly-A+ transcripts were profiled.

**Interested Parties -** This term, for the purposes of this manuscript refers to the range of external stakeholders (e.g., commercial companies, policymakers etc) and rights holders (e.g., Indigenous Peoples) that have an interest in biodiversity genomics research.

**EBP Genome assembly quality standard 6..Q40** **-** Minimum reference standard of 6.C.Q40, i.e. megabase N50 contig continuity and chromosomal scale N50 scaffolding, with less than 1/10,000 error rate.  For species with chromosome N50 smaller than a megabase this will be C.C.Q40. Additional recommendations include K-mer completeness >90%, BUSCO complete single-copy single >90%, BUSCO complete single duplicate < 5%, and Gaps/Gbp <1000.

**Widening Country** **-** Widening countries are countries with low participation rates in FP7 and H2020 projects (low level of investment into research and innovation (R&I)). According to the Horizon Europe [regulation](https://rea.ec.europa.eu/news/eu-committed-research-and-innovation-through-horizon-europe-widening-programme-2022-08-29_en) the Widening countries are: Bulgaria, Croatia, Cyprus, Czech republic, Estonia, Greece, Hungary, Latvia, Lithuania, Malta, Poland, Portugal, Romania, Slovakia, Slovenia and all associated countries with equivalent characteristics in terms of R&I performance and the Outermost Regions.

GDPR - The [General Data Protection Regulation](https://gdpr-info.eu/) (GDPR) was issued by the European Union and became applicable in 2018. The act aims to harmonise the data privacy regulations relating to personal information across Europe. The regulation protects the fundamental rights and freedoms of natural persons and their right to the protection of their data.

**Supplementary References**

**1.** [Dias, E. F. *et al.* Phylogeography of the Macaronesian Lettuce Species *Lactuca watsoniana* and *L. palmensis* (Asteraceae). *Biochem. Genet.* 56, 315–340 (2018).](http://paperpile.com/b/HBikTh/JJYSO)

2. [Pohl, H., Gorb, E. V. & Gorb, S. N. Traction force measurements on male Strepsiptera (Insecta) revealed higher forces on smooth compared with hairy substrates. *J. Exp. Biol.* 223, (2020).](http://paperpile.com/b/HBikTh/XWkK)

3. [Hansen, T. *et al.* The genome sequence of the brown trout, *Salmo trutta* Linnaeus 1758. *Wellcome Open Res* 6, 108 (2021).](http://paperpile.com/b/HBikTh/JJVK)

4. [Laetsch, D. R. & Blaxter, M. L. BlobTools: Interrogation of genome assemblies. *F1000Res.* 6, 1287 (2017).](http://paperpile.com/b/HBikTh/LAKd)

5. [Kathirithamby, J. Host-parasitoid associations in Strepsiptera. *Annu. Rev. Entomol.* 54, 227–249 (2009).](http://paperpile.com/b/HBikTh/Evpc)

6. [Werren, J. H. & Windsor, D. M. Wolbachia infection frequencies in insects: evidence of a global equilibrium? *Proc. Biol. Sci.* 267, 1277–1285 (2000).](http://paperpile.com/b/HBikTh/5Evp)

7. [Hedges, L. M., Brownlie, J. C., O’Neill, S. L. & Johnson, K. N. Wolbachia and virus protection in insects. *Science* 322, 702 (2008).](http://paperpile.com/b/HBikTh/Y2If)

8. [Frumkin, A., Chipman, A. D. & Naaman, I. An isolated chemolithoautotrophic ecosystem deduced from environmental isotopes: Ayyalon cave (Israel). *Frontiers in Ecology and Evolution* 10, (2023).](http://paperpile.com/b/HBikTh/HmtH)

9. [Frumkin, A., Dimentman, C. & Naaman, I. Biogeography of living fossils as a key for geological reconstruction of the East Mediterranean: Ayyalon - Nesher Ramla system, Israel. *Quat. Int.* 624, 168–180 (2022).](http://paperpile.com/b/HBikTh/tA2D)

10. [Gavish‐Regev, E., Frumkin, A. & Na’aman, I. The power of academic and public opinion in conservation: The case of Ayyalon Cave, Israel. *Integrative* (2023).](http://paperpile.com/b/HBikTh/d0HK)
